# Performance of gender detection tools: a comparative study of name-to-gender inference services

**DOI:** 10.5195/jmla.2021.1185

**Published:** 2021-07-01

**Authors:** Paul Sebo

**Affiliations:** 1 paulsebo@hotmail.com, Primary Care Unit, Faculty of Medicine, University of Geneva, Geneva, Switzerland

**Keywords:** accuracy, gender detection, misclassification, name, name-to-gender, performance

## Abstract

**Objective:**

To evaluate the performance of gender detection tools that allow the uploading of files (e.g., Excel or CSV files) containing first names, are usable by researchers without advanced computer skills, and are at least partially free of charge.

**Methods:**

The study was conducted using four physician datasets (total number of physicians: 6,131; 50.3% female) from Switzerland, a multilingual country. Four gender detection tools met the inclusion criteria: three partially free (Gender API, NamSor, and genderize.io) and one completely free (Wiki-Gendersort). For each tool, we recorded the number of correct classifications (i.e., correct gender assigned to a name), misclassifications (i.e., wrong gender assigned to a name), and nonclassifications (i.e., no gender assigned). We computed three metrics: the proportion of misclassifications excluding nonclassifications (errorCodedWithoutNA), the proportion of nonclassifications (naCoded), and the proportion of misclassifications and nonclassifications (errorCoded).

**Results:**

The proportion of misclassifications was low for all four gender detection tools (errorCodedWithoutNA between 1.5 and 2.2%). By contrast, the proportion of unrecognized names (naCoded) varied: 0% for NamSor, 0.3% for Gender API, 4.5% for Wiki-Gendersort, and 16.4% for genderize.io. Using errorCoded, which penalizes both types of error equally, we obtained the following results: Gender API 1.8%, NamSor 2.0%, Wiki-Gendersort 6.6%, and genderize.io 17.7%.

**Conclusions:**

Gender API and NamSor were the most accurate tools. Genderize.io led to a high number of nonclassifications. Wiki-Gendersort may be a good compromise for researchers wishing to use a completely free tool. Other studies would be useful to evaluate the performance of these tools in other populations (e.g., Asian).

## INTRODUCTION

Using tools to infer gender from first name (or first name and surname) can be helpful in medical research, as it is often considered an effective way to save time and resources. For example, using genderize.io, Cevik et al. compared the gender distribution of clinical trial leadership in COVID-19 [1]. They found that only 28% of principal investigators among COVID-19 studies were female, compared to 55% and 42% for breast cancer and diabetes trials over the same period, respectively. In another study, Gottlieb et al. used genderize.io to determine the gender distribution of editorial board members among emergency medicine journals [2]. They found that out of 1,477 editorial board members, only 16% were women.

As suggested by these two examples, one area of research that could particularly benefit from gender detection tools is the study of gender inequalities, whether in terms of scientific publications or citations, grant allocations, or salaries and career advancement processes. Although the number of female physicians exceeds that of their male counterparts early in their careers, their number declines over the course of their medical career, as we recently showed in Switzerland [3]. This phenomenon has been called the “leaky pipeline” [4]. In addition, compared to their male counterparts, female researchers generally receive lower salaries [5, 6] and less funding for their studies [7]. With regard to scientific production, a number of articles also show a clear and persistent gender imbalance in first and/or last authorship to the detriment of females [8–10], and, in general, articles published by female researchers are cited less often than those of their male colleagues [11]. One mechanism that may contribute to the gender gap in citations is the difference in the extent to which women promote their research compared to men [12].

Gender detection tools (i.e., name-to-gender inference services) have three main advantages. They are fast, cost effective, and can be applied retroactively to large datasets. The algorithms used are, unfortunately, often complex and difficult to understand for nonspecialists. In general, they rely on extensive (often openly available) name repositories and try to refine the results obtained using additional information on the cultural context, mainly the family name or country of origin [13].

With the development of gender detection tools, researchers increasingly tend to use them in their studies to speed up data collection without necessarily justifying the choice of method used or discussing their limitations [14]. These shortcomings may be related to the limited number of studies that analyzed the performance of these tools [13, 15, 16]. Of these studies, only one to our knowledge was peer reviewed [13] and, surprisingly, none of them used databases containing both the name and gender of individuals as obtained through self-identification, instead relying on gender determination mainly through Internet queries. As a result, there was a relatively high risk of gender assignment errors in the databases used in these studies to evaluate the performance of gender detection tools.

The objective of this study was to compare the performance of gender detection tools using several databases of physicians practicing in Switzerland.

## METHODS

### Study population

The study was carried out in Switzerland, a multilingual country (four national languages: German, French, Italian, and Romansch) with 36% non-Swiss physicians (outpatient medicine: 33%, hospital medicine: 40%) [17]. The four most common national origins of the non-Swiss physicians were Germany (53%), Italy (9%), France (7%), and Austria (6%).

The study relied on four databases of physicians (total number of physicians: 6,264; 50.4% female). The first database consisted of 2,183 physicians and 908 trainee physicians affiliated with the University Hospital of Geneva, the largest hospital in Switzerland (around 14,000 employees, 17% of whom are physicians) and one of the largest in Europe. The second database consisted of 207 senior physicians practicing in Swiss university hospitals. The last two databases consisted of community-based physicians (510 physicians in Geneva and 2,456 primary care physicians, pediatricians, and gynecologists in Switzerland). For each physician, we extracted first name, surname, and gender.

A number of physicians were listed in more than one database (i.e., duplicates): 123 physicians in two databases and 5 in three databases. After removing all duplicates except the first occurrence, 6,131 physicians were included in the study (50.3% female). In addition, some first names are more common than others, so the lists contained a number of physicians whose first names were identical. For this reason, we also tested the accuracy of the results with a subsample of our study population in which we removed all duplicates for first names and gender except the first occurrence. This subsample consisted of 3,013 physicians, 53.5% of whom were female.

As the study data were imported from real-life databases, first names and/or surnames were often spelled differently depending on the database considered (e.g., names in upper- or lowercase, names with an acute accent or not, compound names separated by a hyphen or not). The various databases were uploaded to the gender inference services without any prior manipulation of the physicians' names. In particular, we did not change the spelling of the first names in the lists.

### Gender detection tools

We selected the gender detection tools according to three criteria. They had to accept at least one data file format (e.g., Excel, CSV, or TXT), be usable by researchers without advanced computer skills, and be at least partially free of charge. Four tools met these inclusion criteria: three partially free (Gender API [18], free up to 500 requests per month; NamSor [19], free up to 5,000 requests per month; and genderize.io [20], free up to 1,000 requests per day) and one completely free (Wiki-Gendersort [21]). For each gender detection tool examined, the response options for gender inference were female, male, or unknown (i.e., name not found). We did not use any of the additional parameters provided by these services, such as those estimating the quality of inference.

### Origin of physicians' first names

The four datasets included in the study did not provide any information regarding the origin or geographic provenance of physicians' names. Cultural context is, however, an important aspect that can greatly influence the accuracy of the gender inference. We used nationalize.io to predict the most likely nationality of physicians based on their first name. We then grouped the countries according to their main official language if it was one commonly spoken in Western countries (i.e., French, English, Spanish, German, Italian, Portuguese) or if it was Arabic. We classified the remaining European countries into Northern European, Southern European, Western European, and Eastern European countries following World Health Organization classifications. The remaining countries were all in Asia.

### Statistical analyses

We evaluated the gender detection tools by computing four performance metrics [22]. These metrics refer to the confusion matrix that contains six components: ff and mm correspond to correct classifications, mf and fm to misclassifications (i.e., wrong gender assigned to a name), and fu and mu to nonclassifications (i.e., no gender assigned) (Table 1).

**Table 1:** Confusion matrix showing six possible classification outcomes

	Female (predicted)	Male (predicted)	Unknown (predicted)
**Female (actual)**	ff	fm	fu
**Male (actual)**	mf	mm	mu

The four performance metrics were calculated as follows:

errorCoded = (fm + mf + mu + fu) / (mm + fm + mf + ff + mu + fu) errorCodedWithoutNA = (fm + mf) / (mm + fm + mf + ff) naCoded = (mu + fu) / (mm + fm + mf + ff + mu + fu) errorGenderBias = (mf – fm) / (mm + fm + mf + ff)

errorCoded estimates the proportion of misclassifications and nonclassifications (and thus penalizes both types of errors equally). errorCodedWithoutNA measures the proportion of misclassifications excluding nonclassifications. naCoded measures the proportion of nonclassifications. Finally, errorGenderBias estimates the direction of bias in gender prediction (i.e., if the result is positive, the estimated number of women is higher than the actual number).

We also investigated whether the consecutive use of two gender detection tools would reduce the number of nonclassifications by allowing some of the first names not recognized by the first tool to be correctly reassigned using the second tool. To this end, we retrieved the first names not recognized by each of the four gender detection tools. For each of these four subsamples, we documented the number of correct classifications, misclassifications, and nonclassifications obtained with the other three tools. We then computed the same performance metrics described above. We performed all analyses with STATA version 15.1 (College Station, TX, USA).

### Ethical considerations

Since this study did not involve the collection of personal health-related data, it did not require ethical review according to current Swiss law.

## RESULTS

Table 2 presents the confusion matrix and Table 4 summarizes the performance metrics for the four gender detection tools evaluated in the study. These two tables show the data for the entire sample of 6,131 physicians, whereas Tables 3 and 5 present the same data for the subsample of 3,013 physicians obtained after removing all duplicates for first names and gender. For this same subsample of physicians, the list of first names for females misclassified as males is provided in Appendix 1, and the list for males misclassified as females is provided in Appendix 2.

**Table 2:** Confusion matrices for gender detection tools (n=6,131 physicians)

Gender detection tool	Classified as female physicians n (%)	Classified as male physicians n (%)	Nonclassified physicians n (%)
Gender API			
Female physicians	3006 (97.4)	67 (2.2)	12 (0.4)
Male physicians	23 (0.8)	3014 (98.9)	9 (0.3)
NamSor			
Female physicians	3031 (98.2)	54 (1.8)	0 (0.0)
Male physicians	70 (2.3)	2976 (97.7)	0 (0.0)
Wiki-Gendersort			
Female physicians	2832 (91.8)	85 (2.8)	168 (5.4)
Male physicians	43 (1.4)	2895 (95.0)	108 (3.6)
genderize.io			
Female physicians	2519 (81.7)	59 (1.9)	507 (16.4)
Male physicians	17 (0.6)	2529 (83.0)	500 (16.4)

**Table 3:** Confusion matrices for gender detection tools after removing duplicates (i.e., physicians with identical first names and gender) (n=3,013 physicians)

Gender detection tool	Classified as female physicians n (%)	Classified as male physicians n (%)	Nonclassified physicians n (%)
Gender API			
Female physicians	1551 (96.2)	49 (3.0)	12 (0.8)
Male physicians	14 (1.0)	1379 (98.4)	8 (0.6)
NamSor			
Female physicians	1564 (97.0)	48 (3.0)	0 (0.0)
Male physicians	44 (3.1)	1357 (96.9)	0 (0.0)
Wiki-Gendersort			
Female physicians	1421 (88.2)	54 (3.3)	137 (8.5)
Male physicians	30 (2.1)	1303 (93.0)	68 (4.9)
genderize.io			
Female physicians	1173 (72.8)	38 (2.3)	401 (24.9)
Male physicians	16 (1.1)	992 (70.8)	393 (28.1)

**Table 4:** Performance metrics for gender detection tools (n=6,131 physicians)

Gender detection tool	errorCoded	errorCodedWithoutNA	naCoded	errorGenderBias
Gender API	0.0181	0.0147	0.0034	-0.0072
NamSor	0.0202	0.0202	0.0000	0.0026
Wiki-Gendersort	0.0659	0.0219	0.0450	-0.0072
genderize.io	0.1766	0.0148	0.1643	-0.0082

**Table 5:** Performance metrics for gender detection tools, after removing duplicates (i.e. physicians with identical first names and gender) (n=3,013 physicians)

Gender detection tool	errorCoded	errorCodedWithoutNA	naCoded	errorGenderBias
Gender API	0.0276	0.0211	0.0066	-0.0117
NamSor	0.0305	0.0305	0.0000	0.0013
Wiki-Gendersort	0.0959	0.0299	0.0680	-0.0086
genderize.io	0.2815	0.0243	0.2635	-0.0099

Overall, the number of misclassified female physicians was slightly higher than the number of misclassified male physicians (for the entire sample: 265 vs. 153; for the subsample: 189 vs. 104).

For the entire sample, the number of misclassifications was low for all four gender detection tools, ranging from 76 (errorCodedWithoutNA 1.5%) for genderize.io to 128 (2.2%) for Wiki-Gendersort. The number of unclassified physicians was 0 (naCoded 0.0%) for NamSor, 21 (0.3%) for Gender API, 276 (4.5%) for Wiki-Gendersort, and 1,007 (16.4%) for genderize.io. Using errorCoded, which penalizes both types of errors equally, we obtained the following results: Gender API 1.8%, NamSor 2.0%, Wiki-Gendersort 6.6%, and genderize.io 17.7%.

For the subsample of physicians, the percentages of inaccuracies (i.e., misclassifications and nonclassifications) were higher, especially for genderize.io. Using errorCoded, the results were as follows: Gender API 2.8%, NamSor 3.1%, Wiki-Gendersort 9.6%, and genderize.io 28.2%.

The number of misclassifications was relatively well balanced between male and female physicians in both samples. errorGenderBias ranged from 0.3% to 0.8% in absolute value for the entire sample and from 0.1% to 1.2% in absolute value for the subsample.

Appendix 3 shows that several combinations of gender detection tools were effective in correctly reclassifying first names not recognized by the first tool: Gender API followed by NamSor, Wiki-Gendersort followed by one of the other three tools, and genderize.io followed by one of the other three tools. Of these various combinations, the most effective in minimizing the number of inaccuracies was the use of Gender API followed by NamSor. Among 21 first names not recognized by Gender API, 17 were correctly reclassified by NamSor. However, the most effective reclassification in percentage terms was observed with the use of genderize.io followed by Gender API or NamSor, with 97% of unrecognized first names correctly reclassified by the second gender detection tool. Appendix 4 shows the performance metrics for combinations of gender detection tools. The percentage of inaccuracies was low for all combinations (ranging from 1.5% for Gender API and NamSor to 3.2% for Wiki-Gendersort and genderize.io).

Finally, Table 6 shows the origin of the first names for the entire sample using nationalize.io. This tool was able to assign a country of origin to 5,215 first names in the study (i.e., 85% of the sample), with the most common origins being French-speaking (32%) and English-speaking (14%) countries. The sample in our study consisted mainly of physicians whose first names were from Western countries or countries whose main official language was one of those commonly spoken in Western countries. Indeed, 88% of the first names were from French-, English-, Spanish-, Italian-, German-, or Portuguese-speaking countries or from another European country.

**Table 6:** Origin of physicians' first names (n=6,131 physicians)

Origin	N^1^ (%)
French-speaking country	1679 (32.2)
English-speaking country	751 (14.4)
Spanish-speaking country	404 (7.7)
Asian country^2^	344 (6.6)
Eastern European country	324 (6.2)
Italian-speaking country	288 (5.5)
Western European country^2^	272 (5.2)
Arabic-speaking country	259 (5.0)
German-speaking country	259 (5.0)
Northern European country^2^	220 (4.2)
Southern European country^2^	217 (4.2)
Portuguese-speaking country	198 (3.8)

1 The total number of physicians does not add up to 6,131 because of missing values (no assignments for 916 physicians (14.9%))

2 If not already classified in another group (e.g., the Arabic-speaking country group for some Asian countries)

## DISCUSSION

### Main findings

For the entire sample of 6,131 physicians practicing in Switzerland, the proportion of misclassified physicians was low for the four gender detection tools that met our inclusion criteria (errorCodedWithoutNA between 1.5 and 2.2%). By contrast, the proportion of unrecognized first names varied among tools (naCoded between 0 and 16.4%). Using errorCoded, which penalizes both types of error equally, Gender API (1.8%) and NamSor (2.0%) were the most accurate tools in our study.

### Comparison with existing literature

Few studies evaluated the performance of gender detection tools [13, 15, 16], and only one to our knowledge was peer-reviewed [13]. In the peer-reviewed study, Santamaria and Mihaljevic compared five gender detection tools (Gender API, NamSor, genderize.io, gender-guesser, and NameAPI) using a dataset of 7,076 manually labelled names. Unfortunately, as the authors pointed out, there was a relatively high risk of gender assignment errors in their dataset, as gender was determined mainly through Internet queries. Like us, they also showed that Gender API and NamSor were the most accurate tools (errorCoded 7.9% and 12.8%). The difference between these results and the performance observed in our study (errorCoded 1.8% and 2.0%) is probably largely explained by the content of the databases used to compute the metrics, with mainly Western first names in our study compared with roughly 50% Asian first names in Santamaria and Mihaljevic's study. Gender detection tools are often least effective with first names from Asian countries [13].

The proportion of misclassifications was low for the four tools tested in our study (between 1.5% and 2.2%). However, if researchers opt for genderize.io, significant contributions of time and effort will be needed to retrieve the gender of unclassified names (16.4%). Wiki-Gendersort is probably a good alternative for researchers wishing to use an accurate and completely free tool, with little risk of misclassification (errorCodedWithoutNA 2.2%) and relatively few unrecognized names (naCoded 4.5%).

To work around the issue of nonclassifications, we show that it can be useful to combine two gender detection tools. Among the different combinations studied, the most effective was the use of genderize.io followed by Gender API or NamSor. Indeed, 97% of unrecognized first names were correctly reclassified by the second gender detection tool. Interestingly, we found that the percentage of inaccuracies was low for all combinations (ranging from 1.5% to 3.2%). Combining two gender detection tools is therefore a very efficient procedure to improve the quality of gender inference.

The databases used in our study contained a number of physicians with identical first names. We repeated the analyses with a subsample in which each first name was represented only once. We found that the percentages of inaccuracies were higher for the subsample than the full sample, which is a logical finding since duplicates are by definition more common first names and therefore probably more easily recognized by gender detection tools. The differences between the two samples were relatively small for Gender API and NamSor but high for genderize.io (errorCoded: 28.2% vs. 17.7%).

Our study highlights three main types of gender misclassification. The majority of errors concerned unisex first names (also known as epicene or gender-neutral first names). The number of misclassifications of these first names was high even for tools that included the surname in the gender assessment (e.g., NamSor). This was the case, for instance, for the first names Andrea, Claude, and Dominique. The second type of error concerned non-Western first names, particularly of Asian origin (e.g., Anh-Tho, Giang Thanh, and Wei-Ta). Finally, many errors were related to unusual or rare first names (e.g., Joan, Manel, and Michal). Some of these names are also unisex, such as Manel, a diminutive of Emmanuel, which is a male name in Catalan but a female name in Portuguese.

The accuracy of gender determination by current tools can probably be further improved in the future, particularly through the inclusion of many non-Western first names in the databases that these tools use for their development. However, a large proportion of queries will be misclassified regardless due to the relatively large number of unisex first names. An interesting solution to improve the accuracy of the results provided by these tools would be to integrate other assessment techniques, such as direct gender extraction for each tested individual with a unisex first name. This extraction, which would use the individual's first and last name, could be done automatically through visits to various websites and/or social networks.

### Implications for practice

The four tools evaluated in our study have the advantage that they can be used even by researchers with little computer knowledge. Of the four, Gender API and genderize.io are the easiest to use, requiring only the download of a database in Excel or CSV format for Gender API and CSV format for genderize.io. After the file is processed, its enhanced version can be downloaded and saved. Although both Gender API and genderize.io are very simple to use, their performance is not similar. Gender API was the most effective of the four tools evaluated in our study (errorCoded 1.8%), whereas the use of genderize.io leaded to a large number of nonclassifications (naCoded 16.4%).

For NamSor, the most convenient method may be to use a connector (NamSor Custom Connector) with Power BI Desktop, which is a free application from Microsoft. The installation procedure is very well described [23]. NamSor was the second most effective tool in our study (all first names were classified, errorCoded 2.0%).

Finally, Wiki-Gendersort requires installation of the module on a computer and then the use of the file_assign() function to assign a gender to a list of first names in a TXT file [24]. This tool was less effective than Gender API and NamSor due to a relatively large number of nonclassifications (naCoded 4.5%) but was more effective than genderize.io.

### Limitations

The study has some limitations that should be mentioned. It was carried out using databases of physicians practicing in only one country. However, this country is multilingual and multicultural, with a significant number of physicians of foreign origin (36%). Unfortunately, these databases did not contain information on the origin or nationality of the physicians, which would have been useful in assessing how performance results varied according to this sociodemographic variable. From our point of view, as suggested by the analysis of the origin of first names using nationalize.io, the study can be generalized to most Western countries but not, for example, to countries in Asia or the Middle East. It is often with first names from these countries that gender detection tools are most fallible [13].

Determining a person's gender on the basis of their first name raises ethical issues by simplifying the concept of gender [25, 26]. The concepts of sex and gender are not interchangeable, as they differentiate between biological aspects of a person (sex) and their sociocultural roles (gender). The dichotomization of gender risks marginalizing individuals who identify as nonbinary or transgender. It would therefore be preferable to complete the data obtained with gender detection tools by asking for self-identification. This would not only increase the accuracy of the data but would also allow for an approach that is respectful of individuals. However, self-identification requires significant resources and is difficult to envisage in the context of large-scale bibliometric studies.

## CONCLUSION

Four gender detection tools met the inclusion criteria of the study, in that they accepted at least one data file format, were usable by researchers without advanced computer skills, and were at least partially free of charge. Three were partially free (Gender API, NamSor, and genderize.io) and one completely free (Wiki-Gendersort). We found that Gender API and NamSor were the most accurate tools. However, Wiki-Gendersort may be a good compromise for researchers wishing to use a completely free tool. Other studies would be useful to evaluate the performance of these tools in other populations (e.g., Asian and Middle Eastern).

## Data Availability

Data associated with this article are available in the Open Science Framework (https://osf.io/kr2mx/).

## SUPPLEMENTAL FILES

**Appendix 1:** List of female physicians misclassified as male

**Appendix 2:** List of male physicians misclassified as female

**Appendix 3:** Number of first names not recognized by gender detection tools and number of correct classifications, misclassifications, and nonclassifications of these first names after the use of a second gender detection tool

**Appendix 4:** Performance metrics for combinations of gender detection tools
