# Supplementary material for: Performance of gender detection tools: a comparative study of name-to-gender inference services
Source: J Med Libr Assoc. 2021 Jul 1;109(3):414–21. doi: 10.5195/jmla.2021.1185 (PMC8485937; doi:10.5195/jmla.2021.1185)
Supplement: Supplementary file 1 — Appendix 1: List of female physicians misclassified as male [file jmla-109-3-414-s01.docx]

Appendix 1. List of female physicians misclassified as male, after removing duplicates (i.e., physicians with identical first names and gender) (N=189 physicians)

| Gender API (n=49) | NamSor (n=48) | Wiki-Gendersort (n=54) | Genderize.io (n=38) |
| --- | --- | --- | --- |
| AKI | ANAM UMAR | ANAM UMAR | AKI |
| ANAM UMAR | ANH-THO | ANH-THO | ANDREA |
| ANH-THO | Adina-Lorelai | BANI | ANOUCHE |
| ANOUCHE | Amel | BARA | Andrea |
| BANI | Avigael | Claude | BANI |
| BARA | Bayi | Claude Marie | Bayi |
| Bayi | CHAE HYUN | DANIELE | Claude |
| Claude | Claude | EREN | DANIELE |
| DANAI | DIEM-LAN | FLORIE | DOMINIQUE |
| DANIELE | EREN | GYORGI | Dominique |
| DOMINIQUE | GAUD | Gaël | EMINENCE |
| Dominique | GIANG THANH | HAYAM | EREN |
| EMINENCE | GUEKSI BENERIKE | Hong-Diep | GABRIELE |
| EREN | GYORGI | IMEN | GAUD |
| GABRIELE | Gaël | INDRIT | GETSY |
| GAUD | Hairong | KALINKA | IMRANE |
| GIANG THANH | Hong-Diep | KIMIYA | INDRIT |
| GYORGI | IMAN | LAURENCE | JOAN |
| Gaël | IMRANE | LEEN | KARA |
| Gyana | INDRIT | LOU | MANEL |
| IMAN | INSA | LUCKA | MICHAL |
| IMRANE | Jacklean | Laurence | MICHELE |
| INDRIT | KILIJE | Laurence Marie | NIMATULLAH |
| INSA | LAURENCE | MANEL | Phi |
| JOAN | MANEL | MANUELLA | Roane |
| Loan | MAYSSAM ASSEM | MARIE-CLAIRE | Russia |
| MANEL | MEHRAK | MARIE-JOSE | SAJINTH |
| MAYSSAM ASSEM | MICHAL | MICHAL | SHAHAR |
| MICHAL | MINAA SAEED | MINAA SAEED | SHAI |
| MICHELE | NIHED | Marie-Claire | SIDNEY |
| MINAA SAEED | NIMATULLAH | Marie-Jose | SIGIRIYA |
| NIMATULLAH | Nariman | Marie-José | SIMONE |
| Phi | Phi | Marquise Diane | Simone |
| Phong Mai | Phong Mai | My-Huê | TAL |
| Ray Leda | Roane | NIMATULLAH | THIEN-AN |
| Russia | SAJINTH | Nariman | TOM |
| SETHU THAKACHY | SETHU THAKACHY | Nikola Miriam | Wei-Ta |
| SHAHAR | SHAHAR | Phi | Yannick |
| SHAI | SHAI | Phong Mai |  |
| SIDNEY | SIDNEY | Ray Leda |  |
| SIMONE | SIMAN | Ruphin |  |
| SOTOUDEH | SOTOUDEH | SHAI |  |
| Simone | TAL | SIDNEY |  |
| TAL | THIEN-AN | SIMAN |  |
| THIEN-AN | TOM | Salimé-Maria |  |
| TOM | VAN-DAI | THIEN-AN |  |
| VAN-DAI | YASSAMAN | TOM |  |
| Wei-Ta | Yannick | Taïma |  |
| Yannick |  | VALENTINE |  |
|  |  | VAN-DAI |  |
|  |  | Valentine |  |
|  |  | Vana |  |
|  |  | Wei-Ta |  |
|  |  | Yannick |  |
