# Supplementary material for: Performance of gender detection tools: a comparative study of name-to-gender inference services
Source: J Med Libr Assoc. 2021 Jul 1;109(3):414–21. doi: 10.5195/jmla.2021.1185 (PMC8485937; doi:10.5195/jmla.2021.1185)
Supplement: Supplementary file 2 — Appendix 2: List of male physicians misclassified as female [file jmla-109-3-414-s02.docx]

Appendix 2. List of male physicians misclassified as female, after removing duplicates (i.e., physicians with identical first names and gender) (N=104 physicians)

| Gender API (n=14) | NamSor (n=44) | Wiki-Gendersort (n=30) | Genderize.io (n=16) |
| --- | --- | --- | --- |
| ANDREA | ADI | AIMAD EDDINE | ARIA |
| ARIA | ANDRE-DANTE | ANDREA | CAMILLE |
| Andrea | ANDREA | ARIA | CORALIEN |
| CAMILLE | ARIA | Andrea | Djahanguir |
| CORALIEN | ARNI | Anoosh | Ikbel |
| Ikbel | Andrea | CAMILLE | JIANCONG |
| Johanne | CAMILLE | Dahman | Johanne |
| Lilian | CORALIEN | GALLIEN | Lilian |
| NAN | Catalin-Mircea | Hossen | NAN |
| Ngoc Dung | Charly | JANIS | NECHAN |
| SAFA | DANIELE | JOAN | RASTINE |
| SIWAR | DUY-ANH | Johanne | SAFA |
| TEMISAN | Daniele | LEMY | SIWAR |
| VIVIAN | Dominique | Lilian | TEMISAN |
|  | ENEA | MEDDY | Thi |
|  | Edward-Kobina | MENGU MA TOOH | VIVIAN |
|  | HAJO | MICHELE |  |
|  | J. Kamanda | Minh-Chanh |  |
|  | JOAN | Minh-Hiep |  |
|  | Johanne | NICOLA |  |
|  | KIM | Ngoc Dung |  |
|  | Kim Hoang-Nam | PIERRE-AUGUSTE |  |
|  | Lilian | Phuc |  |
|  | Liès | Phuc-Hung |  |
|  | MENGU MA TOOH | SIWAR |  |
|  | MICHELE | Thi |  |
|  | MOOTII | Trieu Minh Hai |  |
|  | Minh-Chanh | VIVIAN |  |
|  | NAN | XIAOWEI |  |
|  | NANA KWABENA | YAEL MATEI |  |
|  | NICOLA |  |  |
|  | Ngoc Dung |  |  |
|  | Ochine |  |  |
|  | PATRICE |  |  |
|  | RASTINE |  |  |
|  | SACHA |  |  |
|  | SAFA |  |  |
|  | SIWAR |  |  |
|  | TEMISAN |  |  |
|  | TRUONG-THANH |  |  |
|  | Thi |  |  |
|  | VIVIAN |  |  |
|  | YAEL MATEI |  |  |
|  | YASHA |  |  |
