# Supplementary material for: Performance of gender detection tools: a comparative study of name-to-gender inference services
Source: J Med Libr Assoc. 2021 Jul 1;109(3):414–21. doi: 10.5195/jmla.2021.1185 (PMC8485937; doi:10.5195/jmla.2021.1185)
Supplement: Supplementary file 3 — Appendix 3: Number of first names not recognized by gender detection tools and number of correct classifications, misclassifications, and nonclassifications of these first names after the use of a second gender detection tool [file jmla-109-3-414-s03.docx]

Appendix 3. Number of first names not recognized by gender detection tools, and number of correct classifications, misclassifications, and nonclassifications of these first names after the use of a second gender detection tool

|  | Number of unclassified physicians (%) | Gender API | NamSor | Wiki-Gendersort | Genderize.io |
| --- | --- | --- | --- | --- | --- |
| Gender API | 21 (0.3) |  |  |  |  |
| Number of correct classifications (%) |  | NA | 17 (81.0) | 0 | 2 (9.5) |
| Number misclassifications (%) |  | NA | 4 (19.0) | 1 (4.8) | 4 (19.1) |
| Number of nonclassifications (%) |  | NA | 0 | 20 (95.2) | 15 (71.4) |
| NamSor | 0 |  |  |  |  |
| Number of correct classifications (%) |  | NA | NA | NA | NA |
| Number of misclassifications (%) |  | NA | NA | NA | NA |
| Number of nonclassifications (%) |  | NA | NA | NA | NA |
| Wiki-Gendersort | 276 (4.5) |  |  |  |  |
| Number of correct classifications (%) |  | 222 (80.4) | 231 (83.7) | NA | 206 (74.6) |
| Number of misclassifications (%) |  | 34 (12.3) | 45 (16.3) | NA | 36 (13.1) |
| Number of nonclassifications (%) |  | 20 (7.3) | 0 | NA | 34 (12.3) |
| Genderize.io | 1007 (16.4) |  |  |  |  |
| Number of correct classifications (%) |  | 981 (97.4) | 977 (97.0) | 946 (93.9) | NA |
| Number of misclassifications (%) |  | 11 (1.1) | 30 (3.0) | 27 (2.7) | NA |
| Number of nonclassifications (%) |  | 15 (1.5) | 0 | 34 (3.4) | NA |
