# Supplementary material for: Performance of gender detection tools: a comparative study of name-to-gender inference services
Source: J Med Libr Assoc. 2021 Jul 1;109(3):414–21. doi: 10.5195/jmla.2021.1185 (PMC8485937; doi:10.5195/jmla.2021.1185)
Supplement: Supplementary file 4 — Appendix 4: Performance metrics for combinations of gender detection tools [file jmla-109-3-414-s04.docx]

Appendix 4. Performance metrics for combinations of gender detection tools, i.e. after the use of a second gender detection tool for nonclassifications (N=6131 physicians)

| Gender detection tool | errorCoded | errorCodedWithoutNA | naCoded |
| --- | --- | --- | --- |
| Gender API / NamSor | 0.0153 | 0.0153 | 0 |
| Gender API / Wiki-Gendersort | 0.0181 | 0.0149 | 0.0033 |
| Gender API / genderize.io | 0.0178 | 0.0154 | 0.0025 |
| NamSor / Gender API | NA | NA | NA |
| NamSor / Wiki-Gendersort | NA | NA | NA |
| NamSor /genderize.io | NA | NA | NA |
| Wiki-Gendersort / gender API | 0.0297 | 0.0265 | 0.0033 |
| Wiki-Gendersort / NamSor | 0.0282 | 0.0282 | 0 |
| Wiki-Gendersort / genderize.io | 0.0323 | 0.0269 | 0.0056 |
| genderize.io / Gender API | 0.0166 | 0.0142 | 0.0025 |
| genderize.io / NamSor | 0.0173 | 0.0173 | 0 |
| genderize.io / Wiki-Gendersort | 0.0224 | 0.0169 | 0.0056 |
